# Supplementary material for: Fine-Tuning of Piezo1 Expression and Activity Ensures Efficient Myoblast Fusion during Skeletal Myogenesis
Source: Cells. 2022 Jan 24;11(3):393. doi: 10.3390/cells11030393 (PMC8834081; doi:10.3390/cells11030393)
Supplement: Supplementary file 1 [file cells-11-00393-s001.zip › cells-1503994-supplementary.pdf]

Fine tuning of Piezo1 Expression and Activity Ensures Efficient Myoblast Fusion during Skeletal Myogenesis

Huascar Pedro Ortuste Quiroga[1*], Massimo Ganassi[3], Shingo Yokoyama[2], Kodai Nakamura[1], Tomohiro Yamashita[1], Daniel Raimbach[4], Arisa Hagiwara[1], Oscar Harrington[4], Jodie Breach-Teji[4], Atsushi Asakura[5], Yoshiro Suzuki[6], Makoto Tominaga[6], Peter S. Zammit[3], Katsumasa Goto[1,2,*]

List of Supplementary Material

Supplementary Figure S1. *Piezo1* is expressed in SC-derived myoblasts throughout myoblast differentiation. *Piezo1* downregulation does not alter the expression of *Piezo2*

Supplementary Figure S2. Piezo1 activation increases myogenic fusion

Supplementary Figure S3. Yoda1-mediated activation of Piezo1 decreases myotube width

Supplementary Figure S4. *Piezo1* knockdown reduced f-actin intensity in EDL- and SOL-derived myotubes

Supplementary Figure S5. Different *Piezo1* siRNA targets continue to show decrease in myoblast fusion

Supplementary Figure S6. *Myomaker* is upregulated in response to Piezo1 activation

Supplementary Figure S1 - *Piezo1* is expressed in SC-derived myoblasts throughout myoblast differentiation. *Piezo1* downregulation does not alter the expression of *Piezo2*

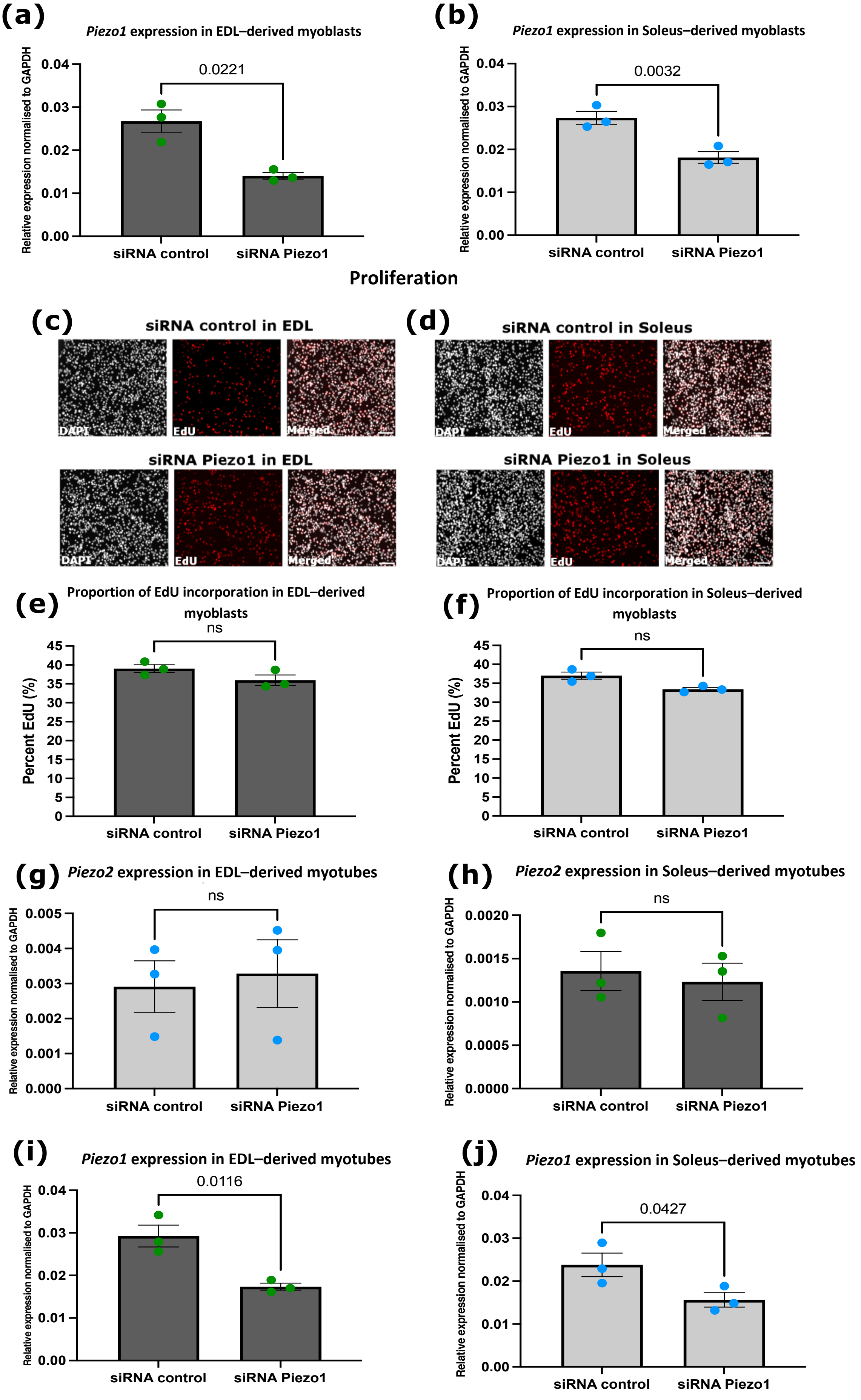

(k)

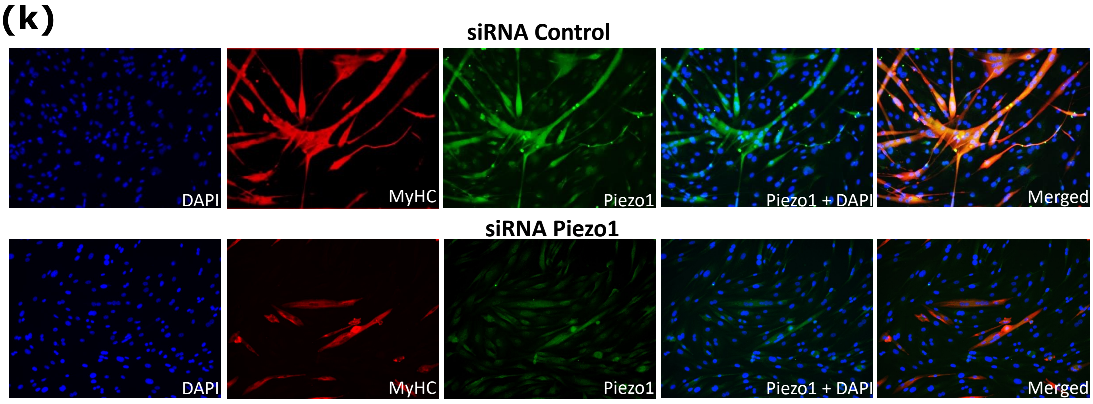
siRNA Control
DAPI
MyHC
Piezo1
Piezo1 + DAPI
Merged
siRNA Piezo1
DAPI
MyHC
Piezo1
Piezo1 + DAPI
Merged

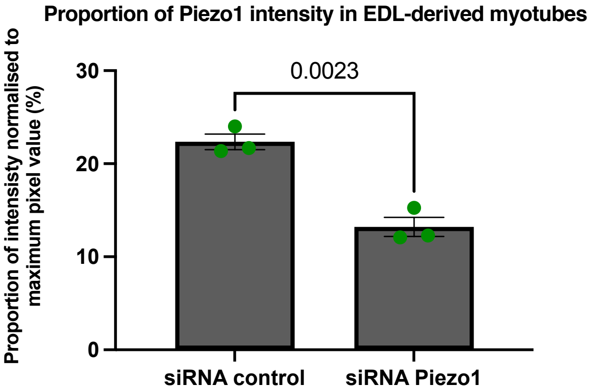
Proportion of Piezo1 intensity in EDL-derived myotubes
Proportion of intensisty normalised to maximum pixel value (%)
30
20
10
0
0.0023
siRNA control
siRNA Piezo1

(l)

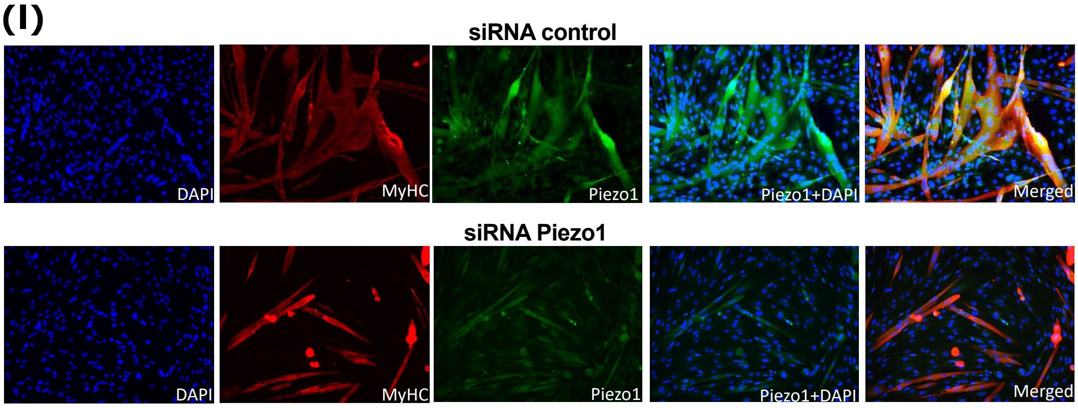
siRNA control
DAPI
MyHC
Piezo1
Piezo1+DAPI
Merged
siRNA Piezo1
DAPI
MyHC
Piezo1
Piezo1+DAPI
Merged

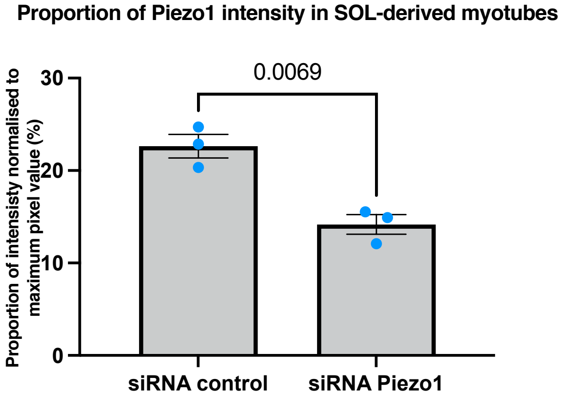
Proportion of Piezo1 intensity in SOL-derived myotubes
Proportion of intensisty normalised to maximum pixel value (%)
30
20
10
0
0.0069
siRNA control
siRNA Piezo1

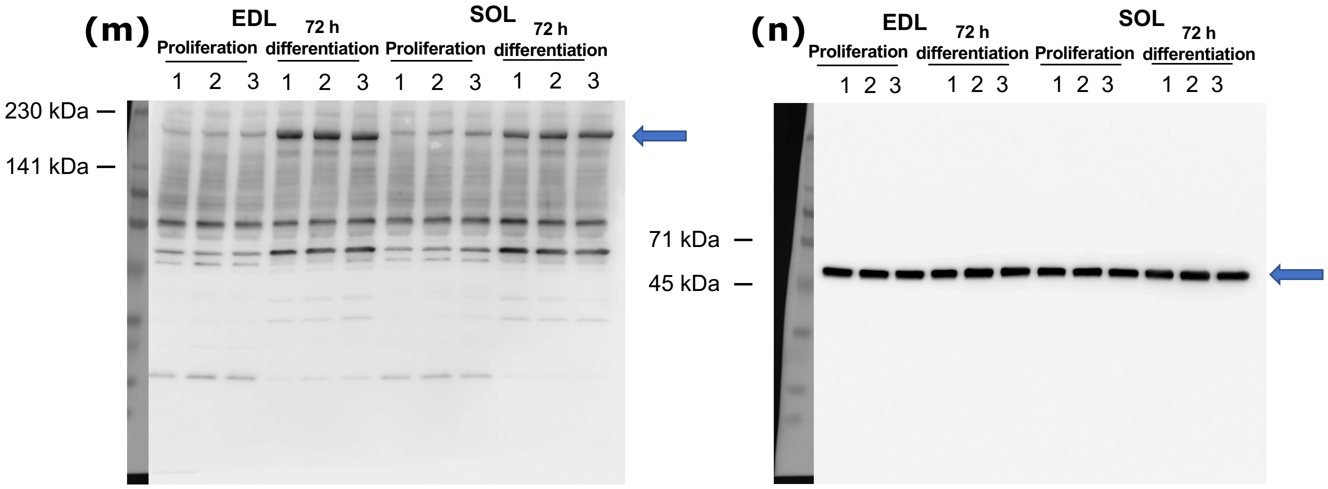

Supplementary Figure S 1. *Piezo1* is expressed in SC-derived myoblasts throughout myoblast differentiation. *Piezo1* downregulation does not alter the expression of *Piezo2*. (**a**) and (**b**) Relative fold changes in expression of *Piezo1* in murine EDL- (dark grey bars, green dots) and SOL-derived myoblasts (light grey bars, blue dots). myoblasts were transfected with 10 nM of either control-siRNA or targeting siRNA against *Piezo1* (Piezo1-siRNA). After overnight incubation, cells were incubated for a further 24 hours and expression of Piezo1 was measured. (**c**) and (**d**) Representative images of EDL and soleus primary-derived myoblasts, transfected with 10nM of siRNA control or siRNA-Piezo1. Following overnight incubation, the medium was changed with fresh proliferation medium and cells were incubated for a further 24 hours, and then subjected to a 2-hour pulse with EdU (red panels). DAPI counterstained nuclei shown in black and white panels. Scale bar is 100 µm. (**e**) and (**f**) Proportion of EdU-incorporated cells relative to total (DAPI) cell count. (**g-j**) Relative fold changes in expression of *Piezo2* (**g** and **h**) and *Piezo1* (**i** and **j**) in EDL- and soleus-derived **myotubes**. Following an initial differentiation period (24 hours at high confluency), cells were transfected with 10 nM of either control-siRNA (siRNA control) or Piezo1-siRNA. After overnight incubation, cells were incubated for a further 24 hours. Values were normalised to *Gapdh*. **(k)** and **(l)** EDL and SOL-derived myotubes were subjected to same conditions as g-j, and immunolabelled for MyHC (red), Piezo1 (green) and counterstained with DAPI. Images taken at x20 magnification. Bar graphs are overall fluorescence intensity of Piezo1 measured by pixel/area in each field of view and expressed as percentages relative to the maximum pixel value. **(m)** and **(n)** original western blot images for Piezo1 **(m)** and β-tubulin **(n)**. Samples are form proliferating myoblasts (24 hr proliferation medium) and differentiated myotubes (72 hr differentiation medium). Numbers above represent individual mice. Data is presented as mean ± SEM from three experiments (n = 3 mice). p values are annotated above graphs showing significance (or ns, not significant) compared to control conditions using a 2-tailed paired student t-test.

Supplementary Figure S2 - Piezo1 activation increases myogenic fusion

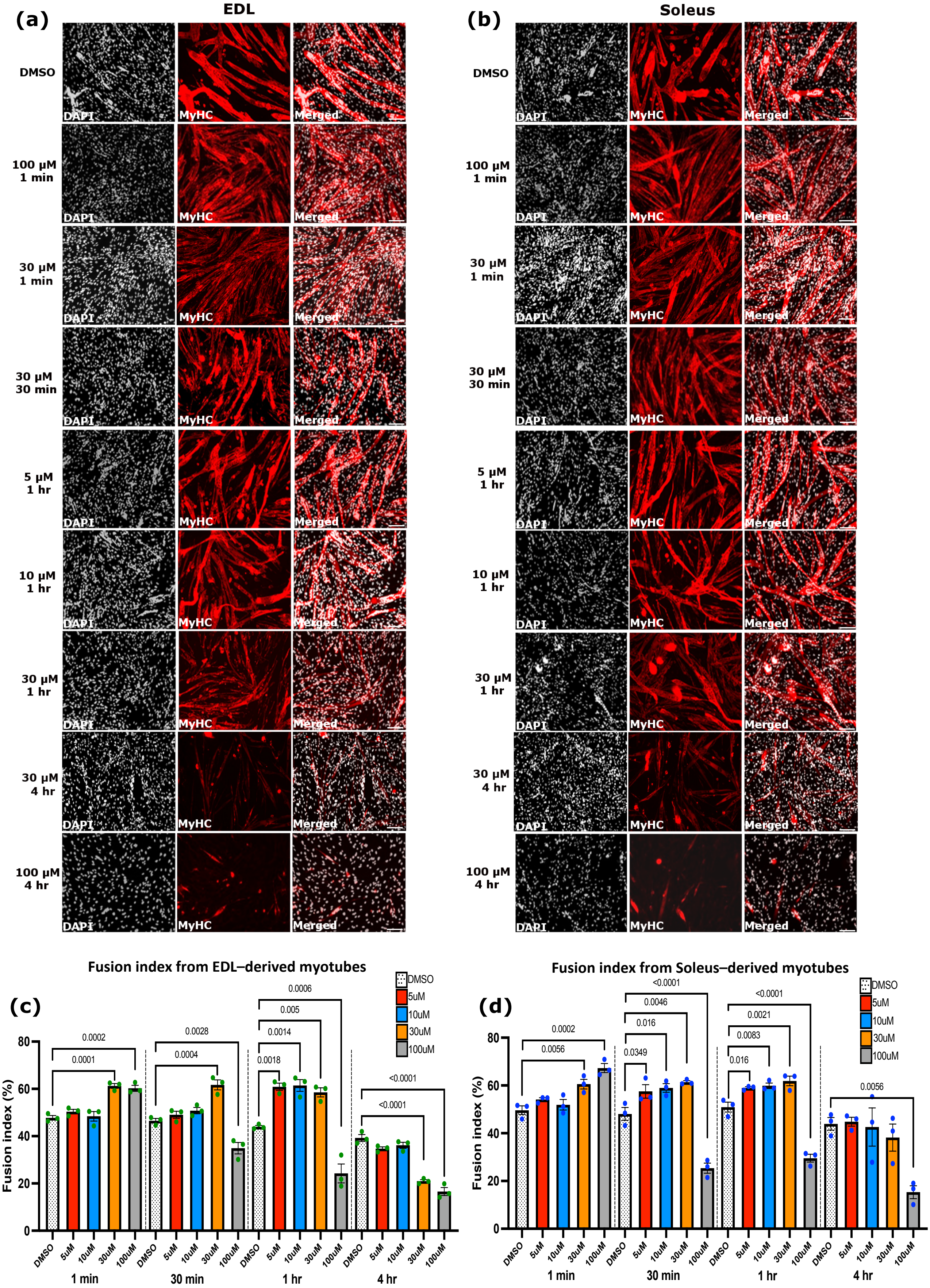

Supplementary Figure S 2. Piezo1 activation increases myogenic fusion. Early forming myotubes were administered with either DMSO (control, white dotted bars) or the Piezo1-agonist Yoda1 at the following concentration: 5 μM (red bars), 10 μM (blue bars), 30 μM (orange bars) and 100 μM (grey bars). Myotubes were incubated for 1 min, 30 min, 1 hour and 4 hours. Following the incubation period, the medium was exchanged with fresh reduced medium (without agonist) and myotubes were incubated for a further 2 days. (**a**) and (**b**) Representative images of cohorts at relevant timepoints and concentrations, immunostained for Myosin heavy (MyHC) (red panels) and counterstained with DAPI (black and white panels). Micrographs taken at x20 magnification. Scale bar is 100 μm. Bar graphs display fusion index in (**c**) EDL and (**d**) soleus-derived myotubes from each time point and concentration variables. Values are mean ± SEM. p values are annotated above graphs showing significance compared to DMSO controls using one-way ANOVA tests followed by the Tukey-Kramer post-hoc. n = 3 mice.

Supplementary Figure S3 - Yoda1-mediated activation of Piezo1 decreases myotube width

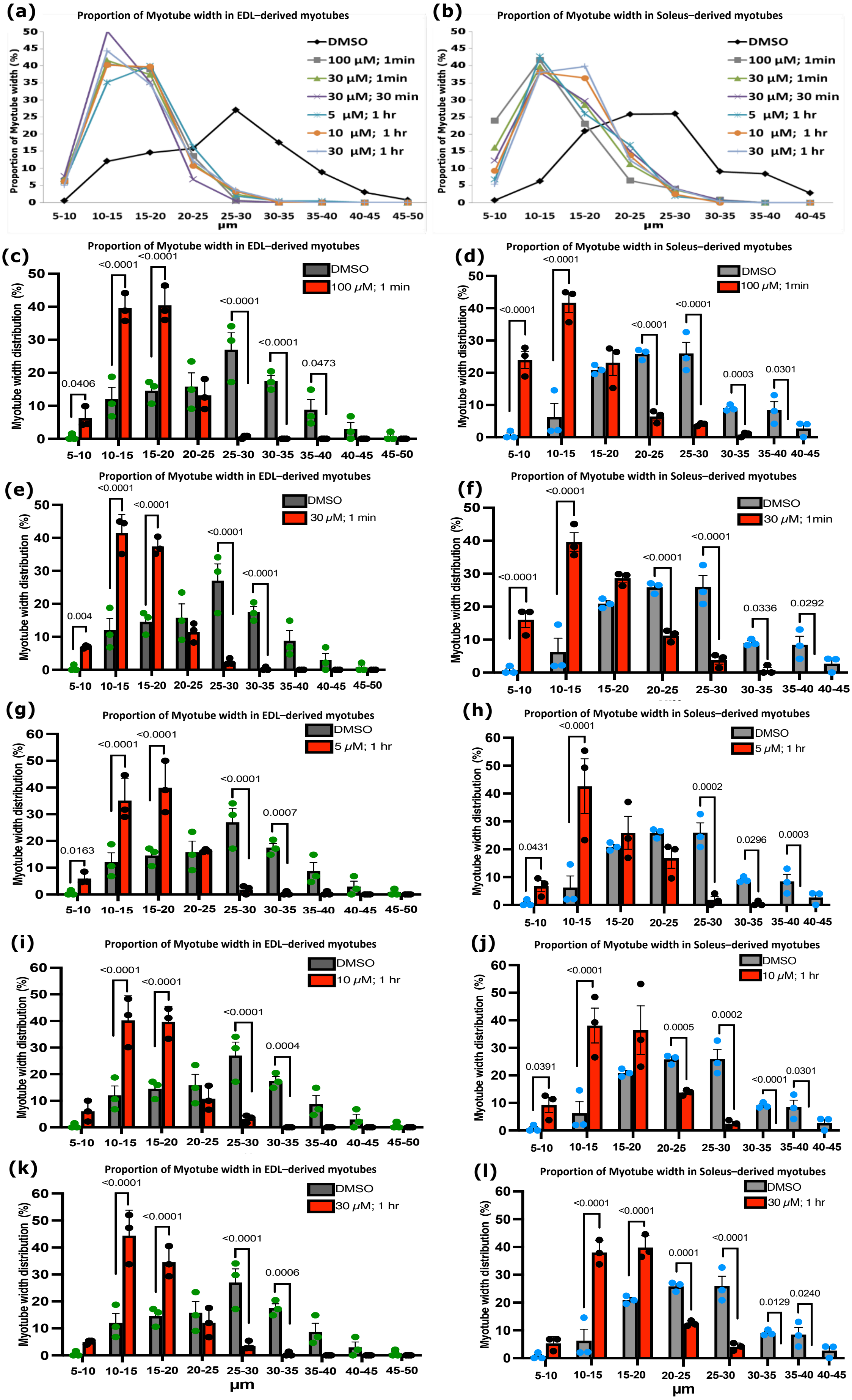

Supplementary Figure S3. Yoda1-mediated activation of Piezo1 decreases myotube width. Myotube width was divided into incremental bins of 5 μm and represented as percentages relative to the total number of myotubes counted. The overall proportion of myotube width distribution in DMSO controls and Yoda1-treated samples is summarised as line graphs in (**a**) EDL and (**b**) soleus-derived myotubes. (**c-l**) Bar graphs comparing DMSO controls and samples which showed increased fusion post Yoda1 treatment (red bars) in EDL (left, dark grey bars green points) and soleus (right, light grey blue points)-derived myotubes. Data is mean ± SEM from three experiments (n = 3 mice). p values are annotated above graphs showing significance compared to DMSO control conditions at each size bin using 2-tailed unpaired student t-test. one-way ANOVA followed by the Tukey-Kramer post-hoc. Please refer to Figure 6 (**a**) and (**c**) for representative images.

Supplementary Figure S4 - *Piezo1* knockdown reduced f-actin intensity in EDL- and SOL-derived myotubes

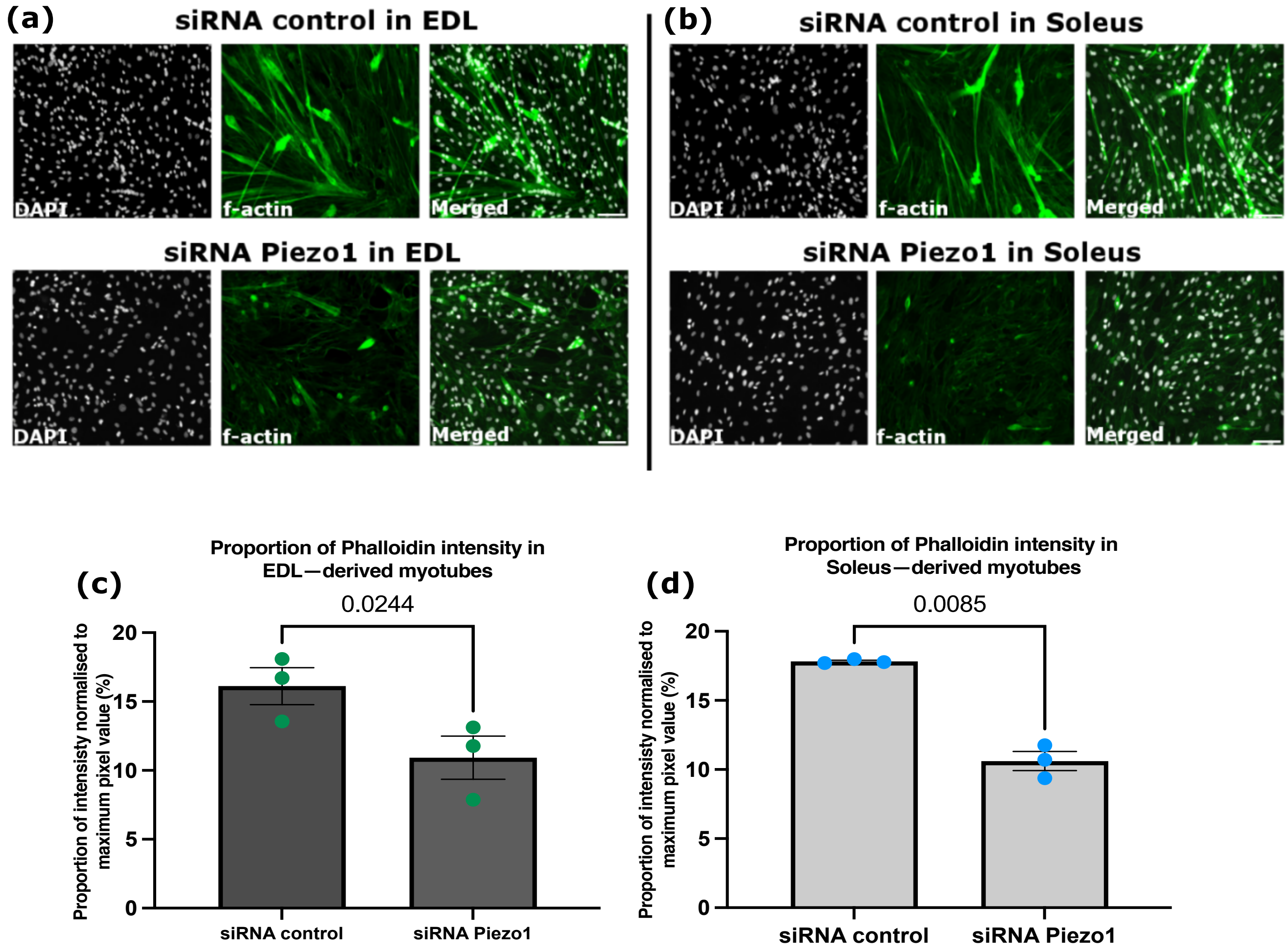

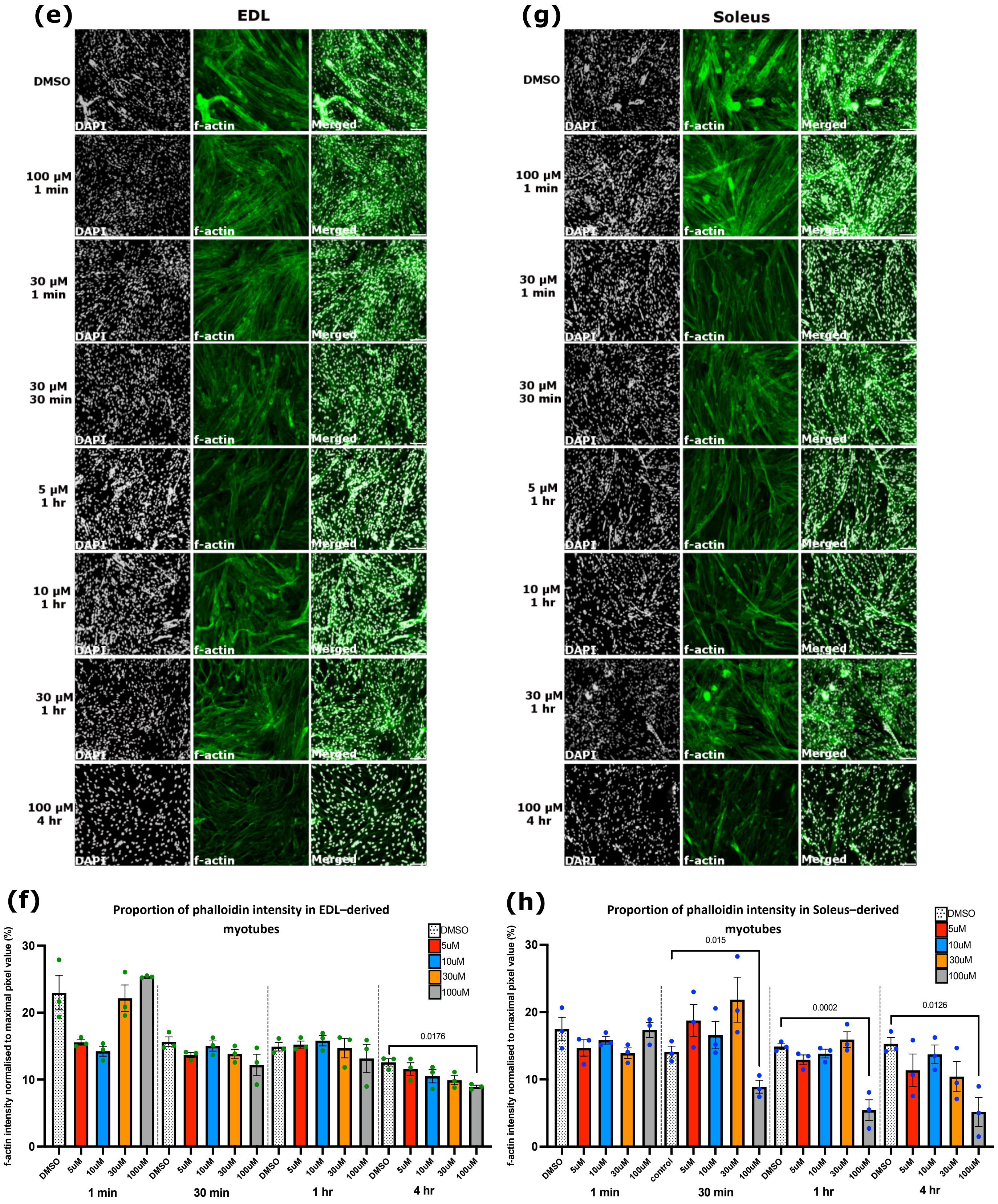
(e)
EDL
DMSO
100 µM 1 min
30 µM 1 min
30 µM 30 min
5 µM 1 hr
10 µM 1 hr
30 µM 1 hr
100 µM 4 hr
DAPI
f-actin
Merged
(g)
Soleus
(f)
Proportion of phalloidin intensity in EDL–derived myotubes
f-actin intensity normalised to maximal pixel value (%)
DMSO
5uM
10uM
30uM
100uM
0.0176
1 min
30 min
1 hr
4 hr
(h)
Proportion of phalloidin intensity in Soleus–derived myotubes
control
0.015
0.0002
0.0126

Supplementary Figure S4. *Piezo1* knockdown reduced f-actin intensity in EDL and SOL-derived myotubes. (**a**) and (**b**) Representative images of EDL and soleus muscle-derived myotubes. Early forming myotubes were transfected with 10nM of control-siRNA (siRNA control) or Piezo1-siRNA (siRNA Piezo1). Following overnight incubation, cells were incubated for a further 72 hours. The cytoskeleton protein f-actin was visualised using fluorescently labelled phalloidin (green panels). Nuclei were counterstained with DAPI (black and white panels). (**c**) and (**d**) Overall fluorescence intensity measured by pixel/area in each field of view (six images per conditions) and expressed as percentages relative to the maximum pixel value. Data is mean ± SEM from three experiments (n = 3 mice). p values are annotated above graphs compared to siRNA control conditions using a 2-tailed paired student t-test. (**e-h**) Early forming myotubes were administered with either DMSO (control, white dotted bars) or Yoda1 at the following concentration: 5 μM (red bars), 10 μM (blue bars), 30 μM (orange bars) and 100 μM (grey bars). Myotubes were incubated for 1 min, 30 min, 1 hour and 4 hours. Following incubation period, the medium was exchanged with fresh reduced medium (without agonist) and myotubes were cultured for a further 2 days. (**e**) and (**g**) Representative images at relevant timepoints and concentrations treated with fluorescently labelled phalloidin (green panels) and counterstained with DAPI (black and white panels). Images taken at x20 magnification. Scale bar is 100 μm. Bar graphs display proportion of f-actin intensity in (**f**) EDL and (**h**) soleus derived myotubes from each time point and concentration variables. Values are mean ± SEM. p values are annotated above graphs showing significance compared to DMSO control conditions using one-way ANOVA tests followed by the Tukey-Kramer post-hoc. n = 3 mice.

Supplementary Figure S5 - Different *Piezo1* siRNA targets continue to show decrease in myoblast fusion

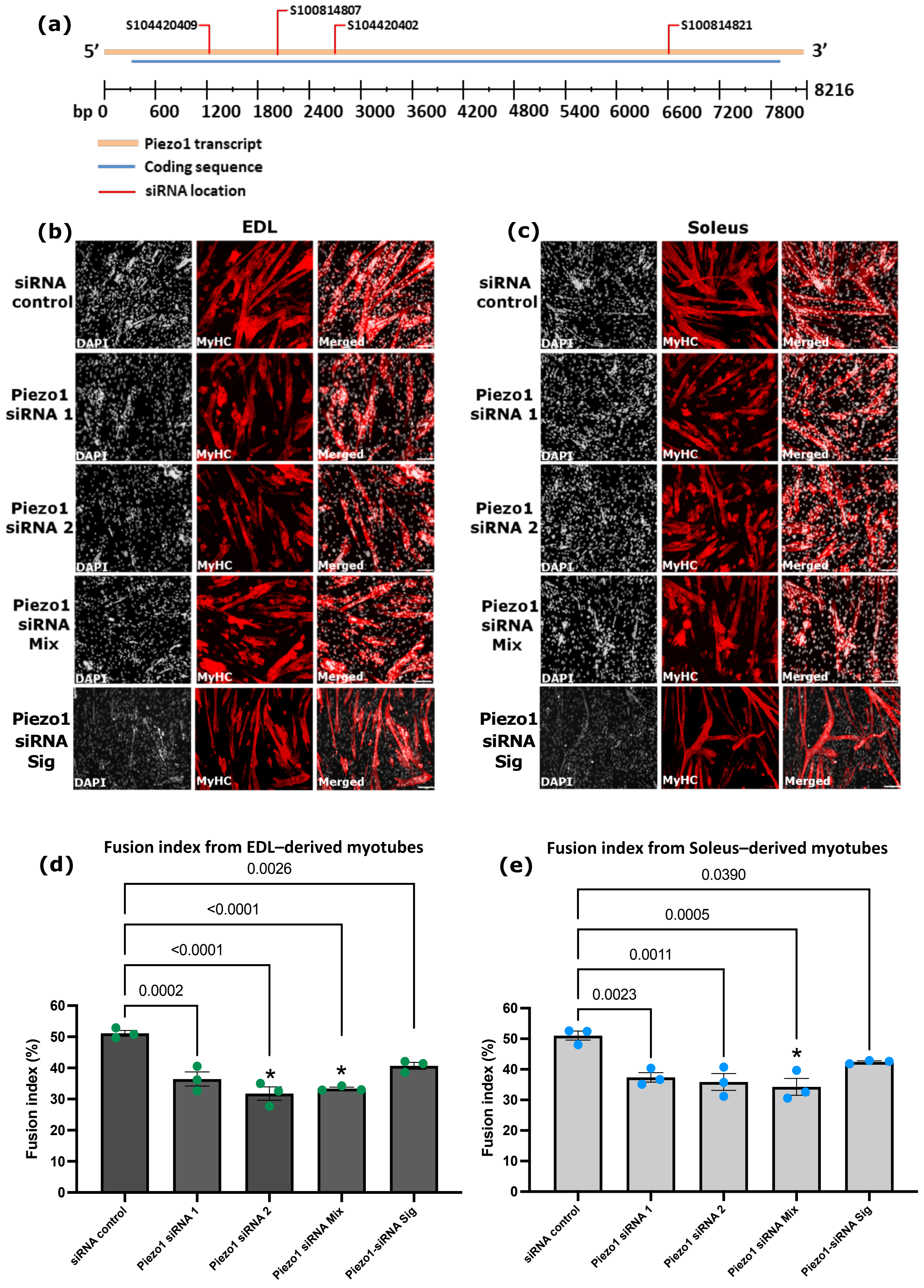

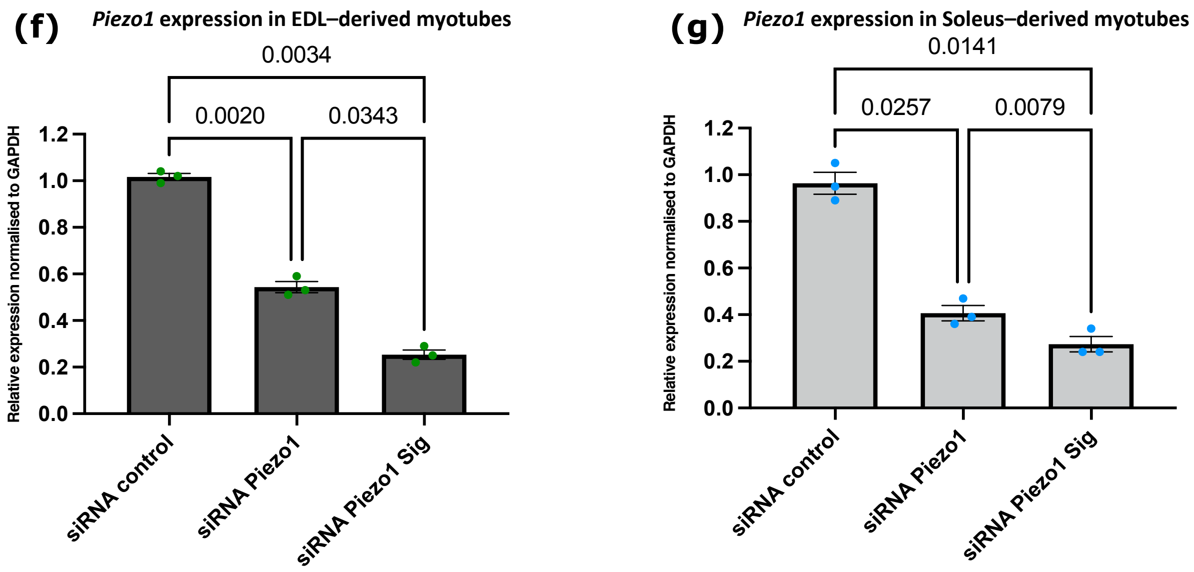

Supplementary Figure S5. Different *Piezo1* siRNA targets continue to show decrease in myoblast fusion. (**a**) Location of siRNAs shown in Table 1 on Piezo1 mRNA. Diagram shows Piezo1 mRNA transcript (orange bar), coding sequence (blue bar) and location of each siRNA (red line). bp: base pair. Diagram is adapted from Qiagen (https://geneglobe.qiagen.com/product-groups/flexitube-sirna). (**b**) and (**c**) Representative images of EDL and soleus muscle-derived myotubes, transfected with 10nM of control-siRNA or different siRNAs specific for *Piezo1* (Piezo1-siRNA 1, Piezo1-siRNA 2 and Piezo1-siRNA Sig from Sigma-Aldrich, SASI_Mm01_00281158). A mixture of four different Piezo1-siRNAs from Qiagen (Piezo1-siRNA Mix) including the one in the main text (Table 2) was also used at 10 nM (2.5 nm each). Myoblasts were transfected and incubated overnight; cells were incubated for a further 72 hours in differentiation medium. Cells were immunolabelled for Myosin heavy chain (MyHC) (red panels) and counterstained with DAPI (black and white panels). (**d**) and (**e**) The fusion index was calculated by counting the total number of nuclei within each myotube (more than two nuclei per myotube) and representing this as a percentage relative to the total number nuclei in the image taken. (**f**) and (**g**) Relative expression of *Piezo1* in (**f**) EDL and (**g**) Soleus derived myotubes transfected with 10nM of siRNA control or siRNA Piezo1 (Qiagen, S104420409) or siRNA Piezo1 Sig (Sigma-Aldrich, SASI_Mm01_00281158). Data is mean ± SEM from three experiments (n = 3 mice). p values are annotated above graphs showing significance compared to siRNA-control conditions using one-way ANOVA followed by the Tukey-Kramer post-hoc. An asterisk (*) denotes significance at $p < 0.05$ compared to Piezo1-siRNA Sig. From table 2, Piezo1 siRNA 1 corresponds to Qiagen, S104420402. Piezo1 siRNA 2 corresponds to Qiagen, S100814807.

Supplementary Figure S6 - *Myomaker* is upregulated in response to Piezo1 activation

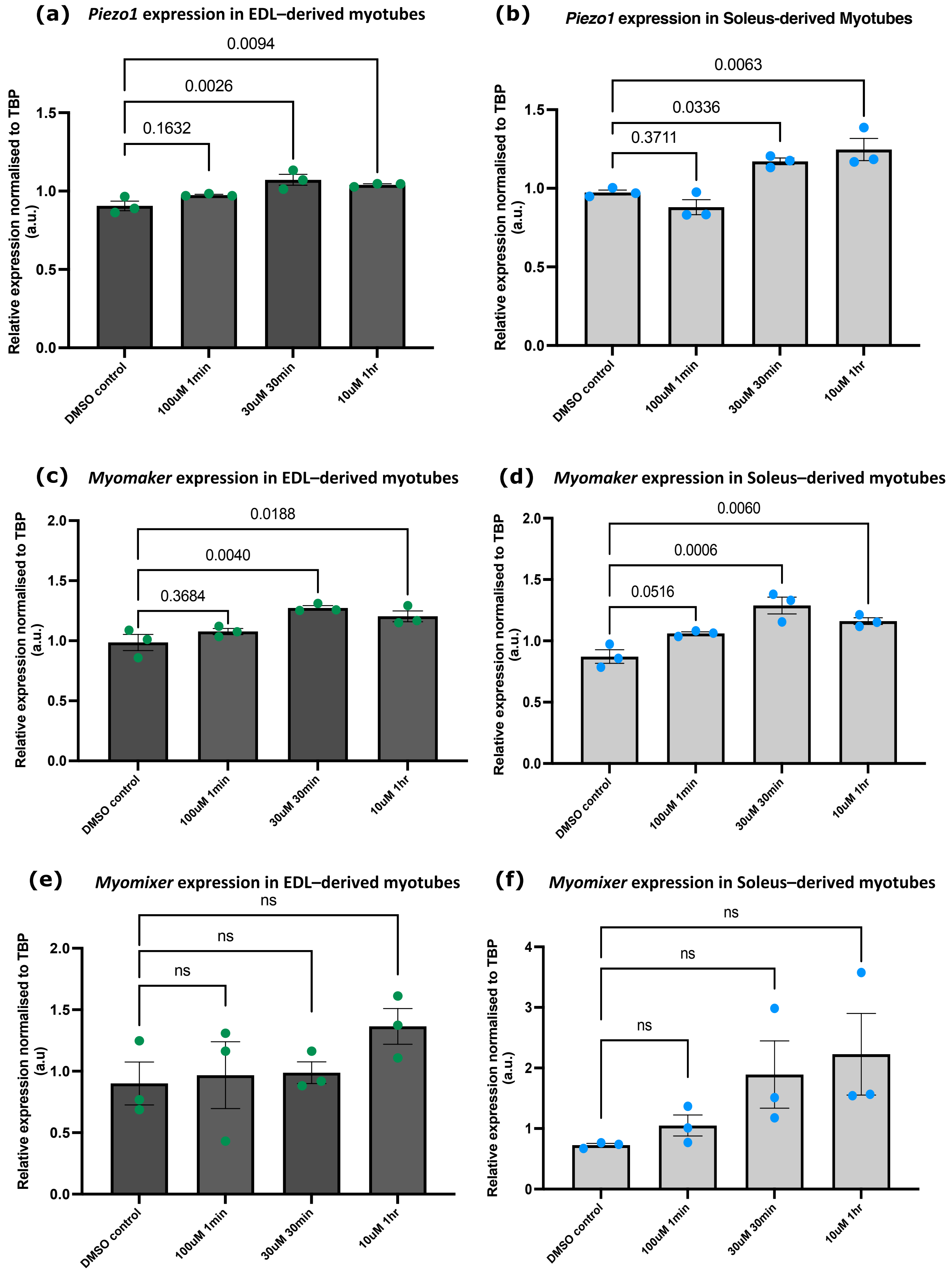

Supplementary Figure S6. ***Myomaker*** **is upregulated in response to Piezo1 activation.** Early forming myotubes were subjected to either DMSO or Yoda1 at 100 μM, 30 μM and 10 μM for 1 min, 30 min and 1 hr respectively. Following incubation, medium was exchanged and myotubes were cultured for a further 2 days. Cells were then collected and RT-qPCRs were performed. (**a**) and (**b**) *Piezo1* expression showed significant increase at 30 μM; 30 min and 10 μM; 1 hr in EDL- and soleus-derived myotubes. Similarly, both (**c**) EDL- and (**d**) soleus-derived myotubes showed increased *Myomaker* expression compared DMSO controls. *Myomixer* on the other hand showed no statistically significant change in expression in (**e**) EDL- or (**f**) soleus-derived myotubes. p values are annotated above graphs showing significance (or ns, not significant) compared to DMSO-control conditions using one-way ANOVA followed by the Tukey-Kramer post-hoc. n = 3 mice.
